# Vitamin D and the vitamin D receptor are critical for control of the innate immune response to colonic injury

**DOI:** 10.1186/1471-2172-8-5

**Published:** 2007-03-30

**Authors:** Monica Froicu, Margherita T Cantorna

**Affiliations:** 1 Department of Veterinary and Biomedical Sciences, Pathobiology Graduate Program, The Pennsylvania State University, University Park, PA 16802, USA; 2 Department of Veterinary and Biomedical Sciences, Center for Molecular Immunology and Infectious Diseases, The Pennsylvania State University, University Park, PA 16802, USA

## Abstract

**Background:**

The active form of vitamin D (1,25(OH)_2_D_3_) has been shown to inhibit development of inflammatory bowel disease (IBD) in IL-10 KO mice. Here, the role of the vitamin D receptor (VDR) and 1,25(OH)_2_D_3 _in acute experimental IBD was probed.

**Results:**

VDR KO mice were extremely sensitive to dextran sodium sulfate (DSS) and there was increased mortality of the VDR KO mice at doses of DSS that only caused a mild form of colitis in wildtype (WT) mice. DSS colitis in the VDR KO mice was accompanied by high colonic expression of TNF-α, IL-1 α, IL-1β, IL-12, IFN-γ, IL-10, MIP-1α and KC. DSS concentrations as low as 0.5% were enough to induce bleeding, ulceration and weight loss in VDR KO mice. VDR KO mice failed to recover following the removal of DSS, while WT mice showed signs of recovery within 5 days of DSS removal. The early mortality of DSS treated VDR KO mice was likely due to perforation of the bowel and resulting endotoxemia. VDR KO mice were hyper-responsive to exogenously injected LPS and cultures of the peritoneal exudates of moribund DSS treated VDR KO mice were positive for bacterial growth. 1,25(OH)_2_D_3 _in the diet or rectally decreased the severity and extent of DSS-induced inflammation in WT mice.

**Conclusion:**

The data point to a critical role for the VDR and 1,25(OH)_2_D_3 _in control of innate immunity and the response of the colon to chemical injury.

## Background

The two forms of inflammatory bowel disease (IBD): Crohn's disease and ulcerative colitis are chronic diseases characterized by aberrant responses to luminal bacteria in genetically susceptible subjects [1]. Although IBD are chronic diseases, the initiation of the inflammation and reactivations of the disease are associated with engagement of the innate immune response and progressive induction of IL-12, IL-1β, and TNF-α in the intestine [2].

The vitamin D receptor (VDR) is a ligand inducible transcription factor that has been shown to be an important regulator of many experimental autoimmune diseases including IBD [3]. A major source of vitamin D results from its manufacture via a photolysis reaction in the skin. Dietary intake of vitamin D is problematic since there are few foods, which are naturally rich in vitamin D. There is mounting evidence for a link between vitamin D availability either from sunshine or diet and the prevalence of IBD [3]. In addition, vitamin D deficiency is common in patients with IBD [4]. Vitamin D is biologically inactive and two hydroxylation reactions occurring in the liver and kidney result in production of active vitamin D (1,25(OH)_2_D_3_). 1,25(OH)_2_D_3_is the form of vitamin D that binds to the VDR and inhibits experimental autoimmunity.

Vitamin D deficiency and VDR deficiency have been shown to exacerbate chronic IBD in IL-10 KO mice [5,6]. Furthermore, treatment of IL-10 KO mice with 1,25(OH)_2_D_3 _resulted in the suppression of IBD symptoms [5]. The effects of vitamin D and 1,25(OH)_2_D_3 _in IBD have begun to be explored and include direct effects of vitamin D on T cells and innate immune cells. Suppression of TNF-α is one mechanism underlying the efficacy of 1,25(OH)_2_D_3 _in vivo [7]. In the gut it is likely that the targets of vitamin D will include epithelial cells, innate as well as acquired immune cells.

Macrophage are important vitamin D targets since they are a potential source of the 1 alpha hydroxylase enzyme (Cyp27B1) that converts the provitamin D hormone 25(OH)D_3 _to active VDR binding 1,25(OH)_2_D_3 _[8]. Macrophage expression of Cyp27B1 has been shown to be increased following TLR ligation in vitro [9]. Macrophage and the TLR pathways are critical regulators of experimental IBD. The TLR-4, TLR-2 and MyD88 KO mice are extremely susceptible to dextran sodium sulfate (DSS) induced colitis [10]. Little is known on whether vitamin D regulates these pathways to maintain gastrointestinal homeostasis in vivo.

DSS initiates mucosal epithelial cell damage by disrupting barrier function, leading to ulceration, and bleeding [11]. A relatively slow mucosal repair process occurs following withdrawal of DSS in wildtype (WT) mice [11]. DSS induced colitis results due to stimulation of the innate immune response since T and B cell-deficient animals such as SCID mice [12], and also SCID mice depleted of NK cells develop DSS colitis [13]. The model is characterized by macrophage production of IL-1β, IL-6, and TNF-α [13]. Macrophage induction at mucosal surfaces are early triggers in an inflammatory cascade that leads to destruction of the intestinal wall.

The role of the VDR and 1,25(OH)_2_D_3 _in regulation of the early innate immune response to DSS was probed in mice. Expression of the VDR was found to be critical to control the innate immune response in the gut. In addition, VDR KO mice had a delayed recovery following DSS withdrawal. 1,25(OH)_2_D_3 _treatments were protective and controlled the early innate response in the colon. VDR KO mice were extremely susceptible to the TLR-4 ligand LPS and furthermore the early death of the VDR KO mice following DSS challenge was associated with bacterial recovery from the peritoneal cavity.

## Results

### Acute DSS colitis is fatal in VDR KO mice

**Figure 1:**
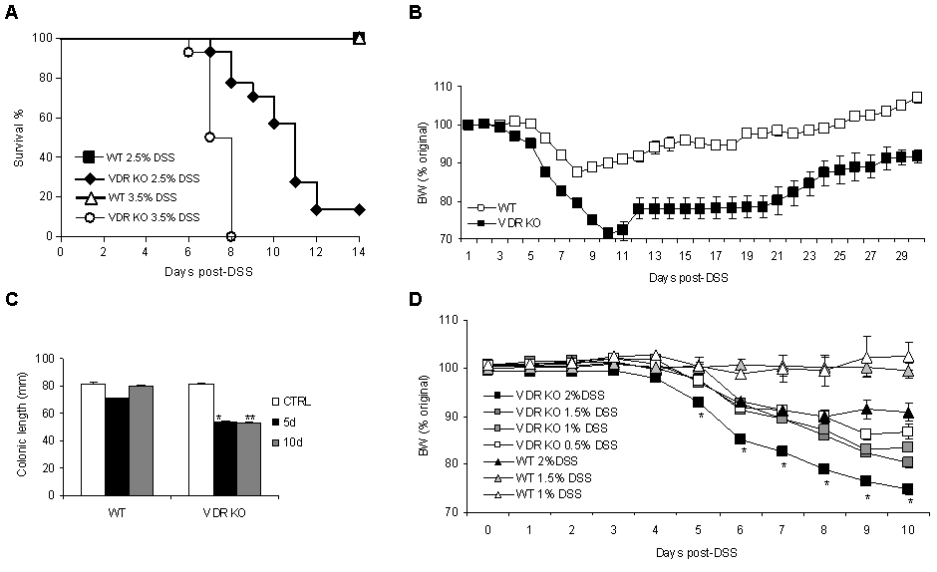
**Increased mortality of VDR KO mice following DSS administration**. **A) **Survival curves in VDR KO and WT mice induced to develop DSS colitis. VDR KO mice died following 2.5% or 3.5% DSS treatment while all of the WT mice survived regardless of DSS concentrations. **B) **BW recovery over time post-DSS treatment. Mice were treated with 2.5% DSS for 5 days. VDR KO mice failed to recover BW following cessation of DSS. **C) **Colon length at day 0 (control-CTRL), day 5 and day 10 of DSS-exposure in WT and VDR KO mice. **D) **VDR KO mice were administered 0.5%–2% and WT mice received 1–2% DSS (n = 5/group). BW changes were monitored, and the values are expressed as the percentage of original BW. *Values from VDR KO mice were significantly different than corresponding values from WT mice. ** WT day 10 colon lengths were significantly longer than WT day 5 colon lengths.

**Figure 2:**
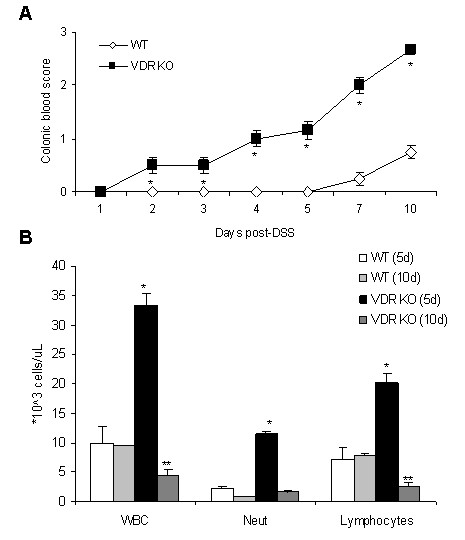
**VDR KO mice have blood in the colon and altered white blood cell values compared to WT mice**. **A) **Colonic blood score as a function of time post-DSS. Two WT and 2 VDR KO mice were sacrificed each day after 2.5% DSS administration and colonic blood score was assessed as described in the Materials and Methods. **B) **Hematological changes in WT and VDR KO mice following 2.5% DSS administration. Error bars represent ± SEM. * WT and VDR KO values were significantly different.

WT and VDR KO mice were induced to develop DSS colitis. At 3.5% DSS the VDR KO mice began to die the 6^th ^day post-DSS administration and by 8 days there was 100% mortality of VDR KO mice (Fig. 1A). Severe diarrhea, bleeding and the loss of more than 25% of the BW preceded mortality of the VDR KO mice. The dosage of DSS was reduced to 2.5% DSS. The mortality of VDR KO (n = 58) mice reached over 80% by day 12 post DSS (Fig. 1A) when the mice lost 25% of their BW (Fig. 2B). At doses of either 2.5% or 3.5% DSS the WT (n = 36) mice did not die and lost only 10% of their BW while showing only mild diarrhea and bleeding.

### Delayed recovery of the VDR KO mice following removal of DSS

In this acute colitis model there is a recovery phase that follows the cessation of DSS [14]. WT mice recovered body weight (Fig. 1B) and the colon lengthened (Fig. 1C) within 5 days of DSS removal as expected. The recovery phase was delayed in surviving VDR KO mice when compared to WT mice as evident by the ultimate inability of VDR KO mice to completely regain BW even 30 days after treatment was stopped (Fig. 1B). Conversely, by day 10 post DSS the WT colons had recovered completely (Fig. 1C) while the VDR KO colons were still significantly shortened and not different than 5 days post DSS (Fig. 1C). Even at lower doses of DSS (1.5%) the recovery period for VDR KO mice was 33 +/- 3.5 days while for WT mice weight was completely regained by 12+/- 1.5 days.

### VDR KO mice are sensitive to very low doses of DSS

Very low doses of (0.5%–2%) DSS that produce little to no harm to the WT intestinal mucosa were tested in VDR KO mice. In VDR KO mice, a dose of 0.5%–1% DSS induced a loss of approximately 15%–18% of the initial BW while this dose of DSS did not affect the BW of WT mice (Fig 1D). A dose of 0.5% DSS in VDR KO mice induced the same decrease in BW as 2.5% DSS in the WT mice (Fig. 1D).

### Hematological and histological alterations in DSS-treated VDR KO mice

**Figure 3:**
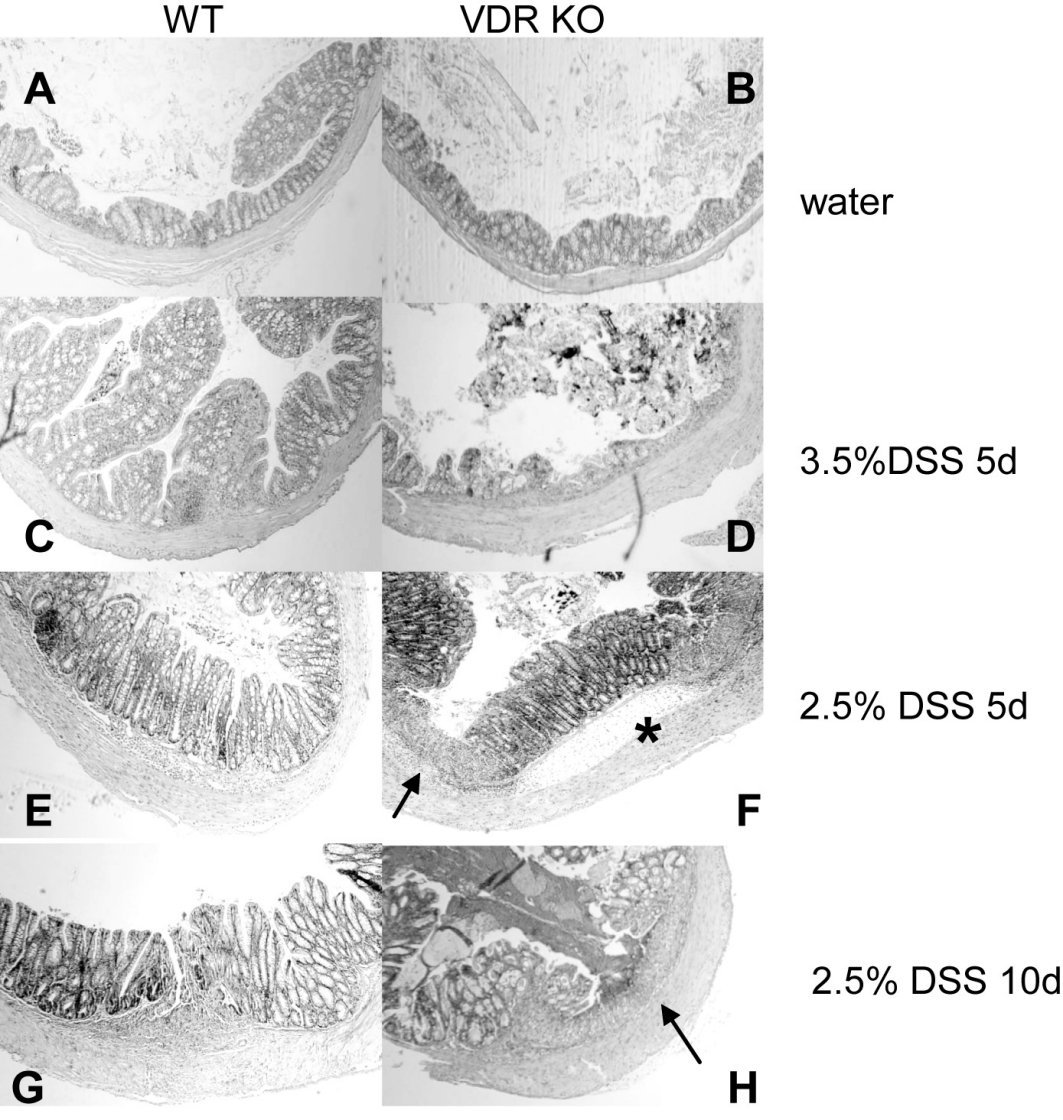
**Colitis development following DSS administration**. Representative sections and histological assessment of colonic samples from **A) **WT and **B) **VDR KO mice receiving water; **C) **WT and **D) **VDR KO mice 5 days after receiving 3.5% DSS; **E) **WT and **F) **VDR KO mice 5 days after 2.5% DSS; **G) **WT and **H) **VDR KO mice 10 days after 2.5% DSS. Edema (asterisk), cellular inflammation in all layers (arrows).

Beginning with day 1 post-DSS administration, blood was detected in the feces of VDR KO mice. Multiple bleeding ulcerations of the intestinal mucosa occurred early and with greater severity in VDR KO mice following DSS administration (Fig. 3). The colon of the VDR KO mice contained blood and scored significantly higher using the colonic blood score than the WT mice (Fig. 2A). Consequently, VDR KO mice became severely anemic; demonstrated by low red blood cells and hematocrit concentrations (data not shown). In addition, the VDR KO mice had increased white blood cell counts, neutrophils and lymphocytes compared to WT mice at 5 d post-DSS (Fig. 2B). Peritoneal exudate fluid was collected and bacterial cultures were made from VDR KO mice that were either treated or not with DSS. Moribund VDR KO mice treated with DSS had positive bacterial cultures (untreated mice were negative) that prove that bacteria was translocating out of the gut and into systemic circulation (data not shown). It is likely that VDR KO mice were dying of severe bleeding and endotoxemia following perforation of the bowel induced by DSS.

**Figure 4:**
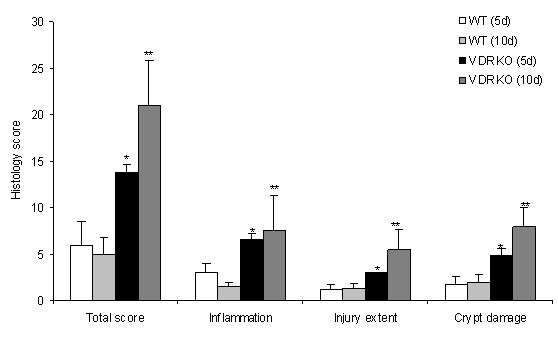
**Histology scores in mice treated with DSS**. Colonic injury score in WT and VDR KO mice that received 2.5%DSS at 5 or 10 days post-administration. Scores were determined as described in Materials and Methods. * VDR KO values were significantly different than WT mice at 5 days post-DSS. ** VDR KO values were significantly different than WT at 10 days post-DSS.

At 3.5 % DSS the VDR KO colon showed a complete destruction of the mucosal surface with most of the epithelium lost and few intact crypts remaining (Fig. 3D). At lower doses of DSS the mucosal damage was less although there were isolated areas where the mucosal surface was absent (Fig. 3F). Both the VDR KO and WT sections showed signs of inflammation and thickening in the outer colonic wall (Fig. 3); however the extent of the damage to the VDR KO mouse was more extensive and failed to resolve. Edema was evident in many of the VDR KO sections (Fig. 3F) and there were significant amounts of blood in the colon after 10 days of DSS but only in the VDR KO sections (Fig. 3H). Blinded scoring of the histopathology sections from VDR KO mice indicate that VDR KO mice had significantly more inflammation, injury and crypt damage than their WT counterparts at both 5 and 10 days post 2.5% DSS administration (Fig. 4, *P < 0.05).

### Over-expression of many cytokines in the colon of VDR KO mice treated with DSS

**Figure 5:**
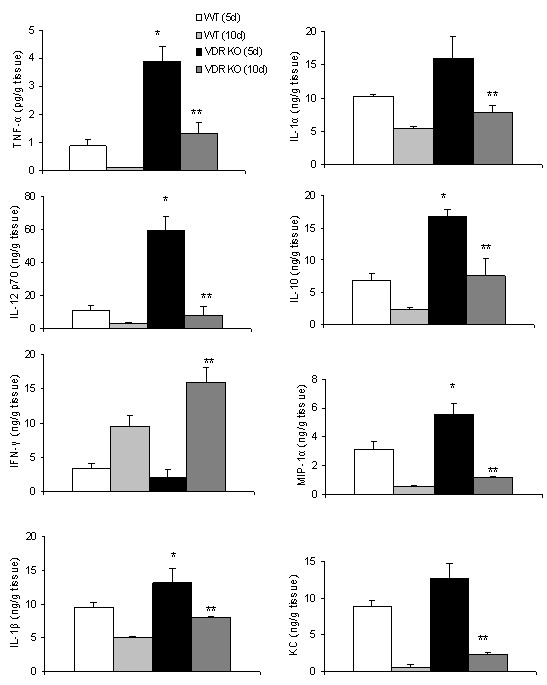
**Cytokine and chemokine production after DSS-induced colitis**. TNF-α, IL-12, IFN-γ IL-1α, IL-1β, IL-10, MIP-1α, KC protein expression in colonic homogenates. * VDR KO values were significantly different than WT mice at 5 days or ** 10 days post-DSS.

Colonic extracts from VDR KO mice treated with DSS showed high levels of TNFα, IL-12, IL-10, IL-1α and IL-1β (Fig 5). The levels of these cytokines were significantly higher in VDR KO mice compared to WT mice (Fig. 5). By 10 days post-DSS treatment WT mice had very little IL-10, IL-1α, and IL-1β and no detectable levels of IL-12, and TNF-α while the levels in the VDR KO colons remained elevated even though the mice had been fed water for 5 days (Fig. 5).

Chemokines recruit neutrophils, macrophage and other effector immune cells to the site of injury. While, the colons of WT and VDR KO mice produced undetectable levels of KC-1, and MIP-1 prior to DSS administration (data not shown); KC-1 and MIP-1 were detectable 5 days after the administration of DSS (Fig. 5) in the colon of both the VDR KO and WT mice. MIP-1 was significantly higher at 5 and 10 days post DSS treatment in VDR KO mice compared to WT mice (Fig. 5). KC production was increased to a similar level in colonic extracts from VDR KO and WT mice 5 days after DSS treatment (Fig. 5) and then decreased in both mouse types by day 10. However, at day 10 VDR KO mice had significantly higher levels of KC in the colon compared to WT mice (Fig. 5).

### Lethal endotoxemia in VDR KO mice

**Figure 6:**
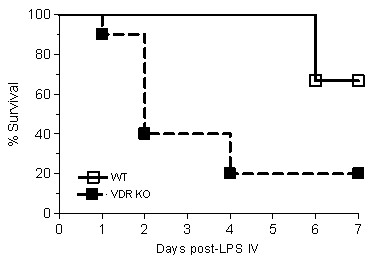
**LPS induced endotoxemia**. The survival of VDR KO (n = 11) and WT (n = 10) mice injected i.v. with 10 mg/kg LPS.

The increased lethality of VDR KO mice following DSS-administration may be a result of endotoxemia following perforation of the large intestine. VDR KO mice (n = 11) and WT (n = 10) mice were injected with the bacterial outer wall component LPS and survival was assessed daily for 7 days (Fig. 6). Shortly after intravenous administration of LPS VDR KO mice became extremely ill, experiencing diarrhea, hunched posture, shaking and lethargy. Similar results were seen when VDR KO mice were injected ip using the same doses of LPS. Eighty percent of VDR KO mice died during the first 4 days post-injection and only 20% survived to day 7. In contrast, 70% of the WT mice survived through day 7 after LPS injection (*p *< 0.001). PBS-injected VDR KO (n = 3) and WT (n = 3) mice remained healthy throughout the 7-day study (data not shown).

### 1,25 (OH)_2_D_3 _improves symptoms of DSS colitis in WT mice

**Figure 7:**
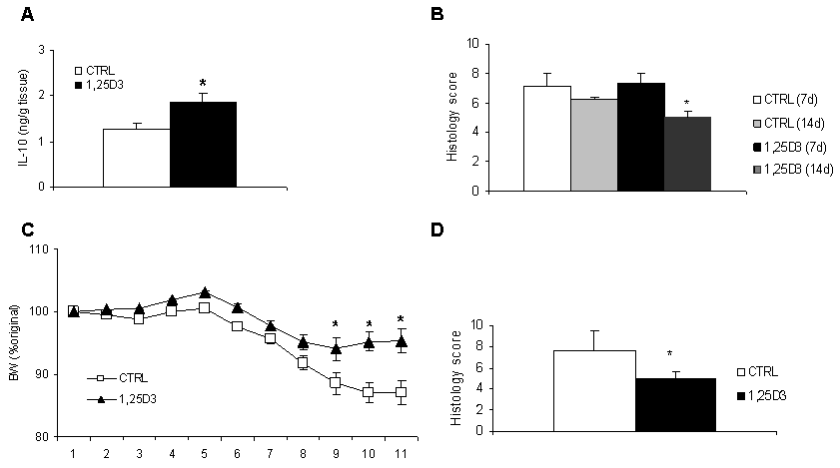
**1,25(OH)_2_D_3 _ameliorates DSS colitis**. **A) **IL-10 production from the colonic homogenates of control (CTRL) or 1,25(OH)_2_D_3 _(1,25D3) fed mice. **B) **Total histological score was determined for control and 1,25D3 fed mice. **C) **Percent weight change of CTRL (n = 6) or 1,25D3 (n = 10) treated WT mice when the treatment was given rectally every other day. **D) **Colonic injury score in rectally treated CTRL and 1,25D3 mice at 10 days post-DSS. *CTRL and 1,25D3 values were significantly different at the corresponding time points.

Oral administration of 1,25(OH)_2_D_3 _(total dose of 1,400 ng/mouse) had no effect on the wasting associated with DSS induced colitis in WT mice (data not shown). However, the 1,25(OH)_2_D_3 _feeding significantly up-regulated IL-10 production in colonic homogenate at 14 days post-DSS induction (Fig. 7A) and this was associated with decreased total histopathological score (Fig. 7B). There was no difference in other cytokines (IL-1, TNF-α or IL-12) measured in the colonic homogenate between 1,25(OH)_2_D_3_-fed and control mice (data not shown). The calcium level in the blood of 1,25(OH)_2_D_3 _fed mice and controls were not different at the end of the experiment (data not shown). 1,25(OH)_2_D_3 _(10 ng, 10 IU) was administered intra-rectally so that delivery of the 1,25(OH)_2_D_3 _dose was to the site of inflammation (colon). The 1,25(OH)_2_D_3 _treated (1,25D3) WT mice shown in Fig. 7C and 7D received a total of 600 ng 1,25(OH)_2_D_3_. Rectal administration of 1,25(OH)_2_D_3 _did not affect serum calcium values (data not shown) while the 1,25(OH)_2_D_3 _treatment prevented body weight loses (Fig. 7C) and resulted in microscopic improvement in histology scores compared to controls (Fig. 7D). Rectal treatment was more effective than feeding 1,25(OH)_2_D_3 _for decreasing the severity of DSS colitis since the same result occurred using only half the dose rectally.

## Discussion

Absence of the VDR results in mice that are extremely susceptible to chemical injury in the gut. DSS treated VDR KO mice are anemic, have high white blood cell counts, large amounts of blood in the colon, and many inflammatory cytokines expressed at high levels. A breach in the intestinal mucosa would lead to the entry of bacteria into the blood stream followed by an excessive, uncontrolled, systemic inflammation including, at the extreme, septic shock. Masubuchi et. al have shown that endotoxin levels detected in the portal blood of rats treated with DSS were higher than those in control rats [15]. In humans, systemic endotoxemia has been described in ulcerative colitis [16,17] and Crohn's disease patients and shown to correlate positively with disease activity, pro-inflammatory cytokine production and the extent of intestinal ulceration [18-23]. VDR deficient mice are extremely sensitive to intravenous or intraperitoneal administration of LPS, supporting the possibility that VDR KO mice with colitis die due to endotoxemia. In addition, bacteria was cultured from the peritoneal cavities of moribund VDR KO mice following DSS treatment. All of the data support the hypothesis that the early mortality of the VDR KO mice treated with DSS is due to perforation of the gut and resulting endotoxemia.

DSS induced colitis is a chemical that damages the colonic epithelium [24] with subsequent recruitment and activation of inflammatory cells and upregulation of inflammatory mediators [25]. During injury or inflammation, intestinal epithelial cells are rapidly proliferating and this process of mucosal repair and regeneration is critical for gut homeostasis [26]. The VDR is highly expressed in human [27] and mouse [28] colonic mucosa and intestinal epithelial cells [29]. 1,25(OH)_2_D_3 _has been shown to control normal villus and crypt development by regulating proliferation and differentiation of intestinal cells [30]. Furthermore, 1,25(OH)_2_D_3 _is an important regulator of cell growth and differentiation in many tissues including the colon [31]. In DSS colitis most of the pathological changes are localized to the distal colon which is a site of low proliferation of epithelial cells [31]. Others have shown that the baseline proliferative state of colonic eptithelial cells in the crypts of VDR KO mice are elevated and showed increased expression of markers for cycling cells (proliferating cell nuclear antigen and cyclin D1) when compared to WTs [31]. Although increased proliferation in the colon of VDR KO mice might be seen as beneficial, it has been shown that crypts with increased numbers of epithelial cells in cell cycle are more susceptible to radiation-induced injury as determined by their inability to repopulate the crypt [32]. The effectiveness of 1,25(OH)_2_D_3 _in protecting the colon and the heightened susceptibility of the VDR KO gut to DSS colitis is likely due in part to the importance of vitamin D and VDR signaling in control of epithelial growth and proliferation.

Once the mucosal barrier is breached, the submucosa is exposed to a vast pool of luminal antigens, including foods and bacteria, and the innate immune responses are engaged to produce large amounts of cytokines. Analysis of cytokine production by colonic homogenates revealed significant elevation of TNF-α, IL-1α, IL-1β, IL-12p70, IFN-γ and IL-10 in VDR KO mice treated with DSS when compared with WT mice. Human and animal studies support the idea that TNF-α and IFN-γ are important pathological mediators of IBD [33]. In humans with IBD approximately two thirds of the patients responded to anti-TNF-α treatments [34], and in mice the intestinal inflammation was significantly attenuated by anti-IFN-γ and/or anti-TNF-α monoclonal antibodies [35]. The production of IFN-γ, TNF-α, IL-1α and IL-1β in the colonic homogenates of VDR KO mice was substantially higher at 10 days post-DSS than in WT mice consistent with the observed delay in recovery from inflammation in these mice. The prolonged expression of inflammatory cytokines corresponded with the increased susceptibility and delayed recovery of the VDR KO mice.

During experimental colitis members of the α-chemokine family are involved in the recruitment of immune cells and the development of intestinal inflammation. Ajuebor et. al have shown in experimental IBD that colonic KC and MIP-1α expression led to leukocyte recruitment in the gut [36]. Furthermore studies by Banks et. al showed that the expression of MIP-1α correlated with the severity of colonic inflammation in patients with IBD [37]. The increased levels of KC and MIP-1α in the colons of VDR KO suggest that in the absence of the VDR many more inflammatory cells may be recruited to the site of injury and then produce inflammatory cytokines that result in severe and fatal form of colitis.

1,25(OH)_2_D_3 _treatments both in the food or locally reduced colitis symptoms in WT mice. 1,25(OH)_2_D_3 _treated WT mice had increased IL-10 production that might serve to inhibit other cytokine responses and lead to a dampening of the cytokine storm in the colon. Furthermore 1,25(OH)_2_D_3 _has the ability to directly induce antimicrobial gene expression and activity of antimicrobial peptide CAMP and defensin β2 genes [38]. CAMP is a potent antisepsis agent that blocks macrophage induction, enhances the survival of mice treated with lethal doses of LPS [39] and accelerates epithelial wound healing [40]. The induction of CAMP and other antimicrobial genes suggested that 1,25(OH)_2_D_3 _might be protective against sepsis after injury and might accelerate epithelial wound healing [38].

## Conclusion

A model develops where the 1,25(OH)_2_D_3 _that is either produced or administered locally in the colon increases epithelial cell resistance to injury and suppresses innate immune responses to luminal antigens through VDR signaling. In the absence of the VDR inflammation in the gut is amplified, colonic epithelial cell proliferation is unregulated and the host fails to adequately maintain gastrointestinal integrity following chemical insult. The data identify vitamin D as a key regulator of gastrointestinal homeostasis and an important player in regulation of the innate immune response.

## Methods

### Mice

Weight (20–25 g) and sex matched 10–12 week old C57BL/6 WT and VDR KO (C57BL/6, gift from M. Demay, Harvard University, Cambridge, MA) were bred for use at the Pennsylvania State University (University Park, PA). All procedures were reviewed and approved by the Pennsylvania State University Institutional Animal Care and Use Committee.

### Induction of colitis

Mice were administered 0.5%–3.5% DSS (MW = 40 kDa; ICN Biomedicals, Aurora, OH) dissolved in filter-purified and sterilized water ad libitum for 5 days, after which the mice were resumed on water for the remainder of the experiment. Animals were weighed daily and monitored clinically for rectal bleeding, diarrhea, and general signs of morbidity. Moribund mice or mice that had lost more than 25% of their body weight were sacrificed and listed as dead following induction of DSS colitis.

### Colitis symptoms

The gross colonic blood scoring system previously described by Siegmund et al[41] was used. The colonic bleeding score was as follows: 0- no visible blood in the entire colon, 1-blood detected in less than 1/3 of the colon, 2- blood detected in less than 2/3 of the colon, 3- blood visible throughout the entire colon. The entire colon from cecum to anus was removed and the length was measured and reported as colonic length as described [11].

The distal colon were removed from the mice, fixed in 10% formalin and sent to the Pennslyvania State University Animal Diagnostic Laboratories (University Park, PA) for H&E staining. Histological analysis was performed blinded by 2 independent investigators on a scale from 0 to 40 as follows: severity of inflammation (0–3: none, slight, moderate, severe), extent of injury (0–3: none, mucosal, mucosal and submucosal, transmural), and crypt damage (0–4: none, basal 1/3 damaged, basal 2/3 damaged, only surface epithelium intact, entire crypt and epithelium lost). Each score was then multiplied by a factor equivalent with the percentage of tissue involvement (× 1: 0–25%, × 2: 26–50%, × 3: 51–75%, × 4: 76–100%)[42].

### Peripheral blood analysis

Blood was collected by cardiac puncture in tubes coated with EDTA (Becton Dickinson Vacutainer System, NJ) and analyzed using an ADVIA 120 Hematology System (Bayer Diagnostic, NY). For some experiments 100–200 μl of blood was collected by retroorbital sinus bleeding using heparinized microcapillary pipettes.

### Colonic homogenate

The distal colon was weighed and the same amount of tissue was cut open and washed in 1XPBS containing penicillin (100 U/ml) and streptomycin (100 μg/ml). Tissue was then homogenized in 1 ml PBS using a razor blade. The homogenized colon tissue was centrifuged at 10,000 *g *at 4°C for 10 min. Cytokine concentrations were determined in the supernatant.

### Cytokine and chemokine ELISA

Serum and supernatants were assayed for mouse TNF-α, IL-12p70, IFN-γ, IL-1α, IL-1β, IL-10 production using Ab pairs and standards provided in the BD Pharmingen kits ELISA (San Diego, CA) according to the manufacturer's instructions. For KC and MIP-1α the ELISA kits were from R&D Systems. (Minneapolis, MN). The limits of detection were 31 pg/ml TNF-α, 125 pg/ml IL-12 p70,125 pg/ml IFN-γ, 31 pg/ml IL-1α, 31 pg/ml IL-1β, 31 pg/ml IL-10, 16 pg/ml KC and 31 pg/ml MIP-1α.

### Endotoxic shock

C57BL/6 mice were injected iv or ip with LPS from *Escherichia coli *0111:B4 (Sigma, St Louis, MO) at a dose of 10 mg/kg body weight. Mice were monitored 3–4 times daily during endotoxic shock, and moribund animals were sacrificed.

### 1,25(OH)_2_D_3 _treatment

Mice received 50 ng/daily of 1,25(OH)_2_D_3 _in the diet as described [5] elsewhere 1 week prior and throughout DSS administration. For local treatment, 50 ng of 1,25(OH)_2_D_3 _was dissolved in 20 uL corn oil and administered rectally 1 day prior to DSS administration and every other day thereafter for the duration of the experiment. Control mice received the corresponding amount of ethanol diluted in corn oil.

### Statistical analysis

Statistical analysis was performed using the paired Student's t test and ANOVAs (StatView; SAS Institute, Cary, NC). P values < 0.05 were considered significant. Error bars represent +/- SEM. The log-rank test was used to compare Kaplan-Meier survival curves.

## Abbreviations

**DSS: **dextran sodium sulfate **IBD: **inflammatory bowel disease **KO: **knockout **VDR: **vitamin D receptor **WT: **wild type

## Authors' contributions

MF carried out the studies, participated in the design of the study, performed the analyses and drafted the manuscript. MTC conceived of the study, participated in its design and coordination and helped to draft the manuscript. Both authors read and approved the final manuscript.
